# Animal Signals, Music and Emotional Well-Being

**DOI:** 10.3390/ani11092670

**Published:** 2021-09-11

**Authors:** Charles T. Snowdon

**Affiliations:** Department of Psychology, University of Wisconsin, Madison, WI 53706, USA; Snowdon@wisc.edu

**Keywords:** music, animal communication, perceptual ability, animal well-being, managed care, pets

## Abstract

**Simple Summary:**

Animal signals can convey information about the animal’s state, but these signals can also be used to influence the behavior of others through emotional contagion. Music can influence the emotional state of human listeners and has also been used therapeutically with a variety of captive species including pets. However, the successful use of music to influence the well-being of animals must be based on an understanding of the natural communication signals of the species including the frequency range and tempos of its own communication signals. Furthermore, different types of music can induce different emotional states. In this paper, I review work using music to influence animal emotion, physiology and behavior, and I outline a theory of emotional induction that predicts what types of music stimuli are likely to influence different emotions and behavior. I will illustrate this with some examples of animal-based music. The use of music to influence the emotional well-being of our pets, farm animals and in zoological parks depends on our understanding the communication system of other species and the variety of emotional states that can be induced through different types of music. My goal is to help those managing animal facilities or advising pet owners to be more aware of the issues involved in using music with animals, as well as provide advice to researchers investigating effects of music on animals.

**Abstract:**

Playing music or natural sounds to animals in human care is thought to have beneficial effects. An analysis of published papers on the use of human-based music with animals demonstrates a variety of different results even within the same species. These mixed results suggest the value of tailoring music to the sensory systems of the species involved and in selecting musical structures that are likely to produce the desired effects. I provide a conceptual framework based on the combined knowledge of the natural communication system of a species coupled with musical structures known to differentially influence emotional states, e.g., calming an agitated animal versus stimulating a lethargic animal. This new concept of animal-based music, which is based on understanding animal communication, will lead to more consistent and specific effects of music. Knowledge and appropriate use of animal-based music are important in future research and applications if we are to improve the well-being of animals that are dependent upon human care for their survival.

## 1. Introduction

Students of communication in nonhuman animals have developed two main models for what animals are communicating. The information model [1] states that animals are communicating about their internal states or about events in their environment and, therefore, providing information to recipients that will be valuable to the recipients. Signals may inform others about the caller’s internal state, what the caller may do next, or about the presence of food or potential predators in the environment. The manipulation/management model [2,3,4], in contrast, suggests that animals use communication to manipulate or manage the behavior of recipients. In this view, communicators are attempting to change or manage the behavior of recipients to the benefit of the caller, but not necessarily providing information that benefits the recipient.

These models need not be mutually exclusive since both information and manipulation can be present in an organism’s communication system. However, the manipulation model has been used to predict specific acoustic features that should be effective with inducing behavior change in listeners [4]. Thus, a series of short rapid calls generally have an arousal effect, and long tonal calls have a calming effect. Dissonant or noisy calls induce fear or aggression, whereas harmonic calls induce calm or affiliative behavior.

Clear evidence for the effects of different acoustic structures on inducing behavior has been shown in studies of how humans communicate with nonverbal organisms, both nonhuman animals and babies. McConnell [5] looked at how animal handlers communicated with sheep herding dogs, with horses and other working animals. Across many cultures and linguistic groups, humans used rapid staccato notes with increasing pitch to arouse animals, long, slow descending notes to slow or calm animals, and a short, sharp plosive note to stop an animal’s movement. At the same time, Fernald [6] showed that similar types of sounds, embedded in the prosodic (or musical) contours of speech, were used by parents to communicate with infants of several different cultures with the same outcomes (short upwardly rising speech, led to increased arousal, long slowly descending speech calmed the infants, and a sharp plosive sound inhibited behavior).

These features appear to be auditory inducers of emotion not only in humans but in other species. Since it is unlikely that animal handlers or parents of infants are experiencing the emotions relating to these calls, the best explanation is that they are trying to manage or change the behavior of animals or infants, supporting the management/manipulation view of communication.

Many of these same structures are seen in the emotional features used in human music: short quick notes are arousing, long harmonic notes are calming, dissonance induces feelings of anger or fear and harmonic patterns lead to feelings of calm and relaxation. These emotional components to music have been hypothesized by musicologists and biologists [7,8] to be present in nonhuman species and serve as the original functional origins of music. Neuropsychological studies of brain activity [9] suggest that these emotional structures of music have different and specific effects on the different brain areas associated with processing different emotions.

Music has often been used by pet owners and by those involved in managed care of animals in research environments, zoological parks, and farms. However, the results of published studies have been inconsistent in the effects of music on animals. Thus, it is important to understand the reasons behind these inconsistent effects and to probe at greater depth how and when music can be used to promote the well-being of animals and when it might be detrimental.

I begin by reviewing many of the studies where music has been used in attempts to alter the behavior or physiology of animals in order to attempt a synthesis of what does and does not work. I will suggest two common problems, failure to match music to animal sensory systems and being unaware that different types of music may lead to different emotional reactions. I will then review some studies that show the importance of considering and eliminating these two problems. I will conclude with some ideas about how and why music evolved and how it can be important in managing our own behavior as well as that of other humans, our pets and other animals that are dependent upon humans for their well-being.

## 2. Studies Involving Music and Animal Responses

I conducted a Web of Science search on 16 June 2021 using the terms “music” and “animals” and examined the reference sections of publications to discover additional studies. I present a summary of the results in Table 1. The table summarizes the results from 58 publications ranging from 1989 through June 2021. The table is organized by species with the types of stimuli presented, and the outcomes observed. Studies were included if music or musical tones were presented to animals with outcome measures of preference, discrimination, improved welfare (reduced stress behavior or increased positive behavior), or altered physiological responses (hormones, neurotransmitters, weight). These outcome measures are shown in the table adjacent to the species names.

Several observations can be derived from the table. The authors do not always specify the specific music used but 36 of 58 studies (62%) reported that classical music was among the stimuli used and of these 36 nearly half specified Mozart as the composer (17 of 36). Other genres were occasionally used including Indian, African and Japanese music, Romanian folk music, hard rock, easy listening, country music and jazz. The effects of music often differ within the same species which I will summarize by general taxonomic category.

Among apes, orangutans preferred silence to music [10] and four studies in gorillas played natural forest sounds, with one showing increased calming, another showed increased stereotypic behavior and two showed no change [11,12,13,14]. Rock music had no effect on gorillas and in one study slow tempo classical music reduced anxious behavior [14], whereas two studies recorded no significant effects of classical music [12,13]. Chimpanzees preferred rock music to silence in one study [15]. In another study, instrumental music increased affiliation while vocal music reduced agonistic behavior [16]. Unspecified music was said to reduce aggression and exploration while increasing social grooming and resting [17]. Chimpanzees preferred Indian and African music to silence [18], and a study of a single young chimpanzee reported a preference for consonant over dissonant music [19]. In gibbons, a mix tape of classical music produced no behavioral change in Moloch gibbons [20], whereas a study playing back the animal’s species typical songs led to increased activity in Lar gibbons [21].

In monkeys, oldies radio music led to no change in blood pressure in baboons, but did reduce heart rate and activity [22]. Classical music, along with silence, produced no changes in response times of rhesus monkeys to neutral stimuli versus emotional stimuli, whereas playing noise led to delayed response time to all stimuli [23]. Marmosets and tamarins preferred Mozart to heavy metal, but preferred silence overall [24]. A study of elephants found unspecified classical music reduced behavioral stereotypes [25].

In dogs, classical music increased sleep and rest behavior in three studies, whereas rock music led to increased activity and barking in three studies [26,27,28]. However, two other studies reported no effects of music on behavior [29,30]. Thus, the effect of music on dogs is quite mixed.

Researchers have studied a variety of farm mammals and these also show mixed results. In lambs, “easy listening” music calmed animals but playing noise led to increased weight gain [31]. In cattle, country music led to increased approach to milking stalls, but no other types of music were tested [32]. Ponies showed no significant behavioral change to a variety of musical genres, but there was a nonsignificant tendency toward increased feeding with country music [33]. One study in piglets found no effects of music [34], whereas two other studies in piglets found that fast tempo music increased activity, play, and tail wagging [35,36].

Many studies have been carried out in rodents. Studies involving playing Mozart perinatally to mice and rats improved learning in young even though the music is not in the optimal frequency range of hearing for these animals [37,38]. Possibly the rhythmic pulse of the music could be detected. Mozart’s music decreased blood pressure in hypertensive rats, whereas the 20th-century music of Ligeti increased blood pressure in one study [39] but no differences were seen in another study that compared the music of Bach with 20th-century music by Stravinsky [40]. Photoperiod influenced the effects of music [41]. One study played pure tones and found increased ghrelin levels and weight gain. Six studies on adult rats have played music by Mozart [41,44,45,46,47,48], with three reporting decreased fear, stress and anxiety [41,45,47]; two studies showed increased spatial learning and increased levels of Brain-Derived Neurotropic Factor that facilitates spatial learning [45,46], and one showed decreased blood pressure in hypertensive rats [48]. Interestingly, this last study found that the effects were the same when all frequencies below 4 kHz were filtered out, meaning that most of the music in the range of human communication was irrelevant.

Several studies have looked at the effects of music on poultry. In hens, one study found that music increased stress behavior [49]. In another study, music had no effect, but noise was aversive [50]. In another study, sitar music increased synaptic binding proteins [51], increased calcium binding proteins and increased neuronal density and volume [52], whereas loud noise led to decreased neuronal density and volume [53]. Loud music enhanced auditory function in comparison with loud noise [54] and Mozart’s music reduced stress physiology, but also reduced growth [55]. Chickens preferred consonant to dissonant music [56], a finding seen only in one young chimpanzee in other animal studies [19]. In a review of several studies on chickens, the playing of happy music increased “happy” behavior, whereas playback of sad music increased “sad” behavior. Both types of music decreased anxiety and anger. Happy music also induced behaviors similar to those induced by oxytocin, a hormone thought to be involved in affiliative bonding behaviors. There was also an increase in brain levels of the reward neurotransmitters, dopamine and norepinephrine. The authors of this review had previously done similar studies in rats with no effects of music on behavior and observed that most human-based music was outside the range of natural communication in rats, but not of chickens [57].

In other birds, pigeons could discriminate between Bach and Stravinsky and could generalize this discrimination to other similar composers [58]. Some Java sparrows could learn to discriminate between Bach and Schoenberg and generalize to composers similar to that which was reinforced [59], and some sparrows showed a preference for Bach [60].

Finally, research in fish has shown a variety of effects of music. In carp, both Mozart and Romanian folk music increased growth and reduced stress [61,62]. Mozart also increased growth in seabream [63]; but in goldfish, lute violin music had no effect on growth or weight [64]. Goldfish could discriminate between Bach and Stravinsky but preferred neither [65]. Finally, music presented at different tempos to turbot showed increased growth with slow tempos and impaired growth with fast tempos [66].

In summary, the results of this survey indicate that music has a variety of effects that vary within and between species. There are few consistent results and it would be difficult to find any consistent effects of music, despite the large number of studies. In the next section, I will suggest why this may be the case and suggest some new ways to think about the use of music in animal well-being.

## 3. A Critique of Past Research and Suggested Solutions

There are two main reasons for the failure of music to produce consistent results when played to animals: (1) animal sensory and communication systems differ from our own and vary according to species; (2) music is highly variable both between and within genres. So music must be selected carefully to produce the desired emotional effects. I will consider each of these in turn and then provide some examples of successful development of species—appropriate music in tamarin monkeys and cats.

### 3.1. Matching Sensory Systems

It is widely known that some animals perceive and communicate about the world in very different ways from humans. Some well-known examples are the use of infrasound (frequencies below the range of human hearing) by elephants and many cetaceans (dolphins and whales) and the use of ultrasound (frequencies above the range of human hearing) by bats and many rodents. Thus, it might be obvious that for music to be effective for these species, it would need to be outside the range of human hearing. What is less obvious is that even within the range of human hearing ability, different species may use frequencies very different from what we use for human communication and music (see [67]).

A couple of compelling examples illustrate this. Rats communicate using much higher frequencies than humans and many of their vocalizations extend into the ultrasonic range. Thus, Akiyama and colleagues [48] found that when they filtered out frequencies in the human range (below 4 kHz), playing Mozart to hypertensive rats was just as effective in reducing blood pressure as when the full auditory spectrum was available. The only thing that mattered to rats was whatever was transmitted above 4 kHz. Many inexpensive speakers may not be able to produce the higher frequencies perceived by rats so some failures to find effects in rodents may be due to this. Panksepp and Bernatzky [57] deliberately switched their work from rats to chickens when they found rats unresponsive to the music they played. With chickens, they found clear effects of music on behavior and on brain neurotransmitters.

Cotton-top tamarins, small monkeys from northern South America, preferred Mozart to heavy metal when tested, but preferred silence to Mozart [24]. The authors concluded that these monkeys had no interest in music. However, the natural calls of these monkeys average about three octaves higher than human speech and music, so they may be responsive to music within their frequency range. In Section 3.3, I will describe results using species-appropriate music with this species of tamarins.

Tempo or pulse is often not considered in selecting music, but music with tempos that match the resting heart rate of humans tends to be calming, whereas tempos that match higher heart rates induced by exercise or dance are more arousing [68]. It seems likely that this principle would hold with other species as well and, in general, smaller bodied animals have higher resting heart rates. It is also be important to acknowledge the range of heart rates of another species when determining what tempos are likely to be most effective with that species.

Studies have shown that tempos approaching natural tempos are important. For example, chicks prefer tempos that are most similar to maternal call rate [69] and are attracted more to tempos approaching patterns of natural vocalizations [70]. Some species have shown the ability to match different tempos (cockatoos [71], Java sparrows [72] sea lions [73]). Harbor seals [74] show trajectories toward adult tempos over development. A single bonobo was resistant to entrainment by experimenters, having a preferred rate of drumming [75].

This critique has focused on cases where researchers have not fully considered the sensory abilities of the species they are testing. There are, of course, some other research papers where these points have been considered.

### 3.2. Music Genres Are Not Uniform in Emotional Effects

The second main reason for disparate results is that many researchers have assumed that a specific genre of music has a uniform emotional effect. Thus, classical music is often thought to be relaxing (or boring), with rock music being arousing. However, there is considerable variation within each genre. For example, much of Mozart’s music can be considered upbeat and arousing and the specific pieces used in many of the studies with animals would be arousing to human listeners. At the same time, some classical music can be relaxing and calming, while still other pieces may be able to induce fear or anger. There has been considerable research on how humans perceive emotions in music and Table 2 summarizes several principles that appear to be important in how humans perceive emotions in music. These principles hypothesized by [76] have been validated in experiments with musicians [77], and Western nonmusicians tested with both Western music [78] and unfamiliar Indian ragas [79].

If the work on how humans communicate with working animals and with nonverbal infants can be generalized to other species, then the principles shown in Table 2 should be applicable to other nonhuman species as well. Thus, if one wants to calm an animal (perhaps a recent arrival at a shelter, or an animal in a clinic) then music should be slow, with long notes (legato), with generally descending pitches, relatively low amplitude and harmonic intervals. If one wishes to arouse an animal (such as one showing depression or lethargy), then the tempo should be fast, the changes in pitch generally ascending, with short notes that have a fast attack speed (staccato) and again harmonic intervals. Music that induces fear is usually rapid in tempo, high pitched with staccato notes, loud amplitude and dissonant intervals. Finally, threat is conveyed by a moderate tempo, low dissonant notes, moderate amplitude and dissonant intervals with a fast attack speed. Most people working with captive animals or pets would probably wish to avoid inducing these last two states in animals, but it is important to be aware that the use of these features may possibly induce unwanted behavior. Table 1 gives some examples of unspecified slow music calming animals and faster music arousing animals (see, for example, the entries for pigs).

Since consonant music is hypothesized to be therapeutic for most uses with animals, it is important to consider some additional literature on how animals perceive consonance. A single chimpanzee was shown to prefer consonant to dissonant music [19] and marmosets and tamarins preferred the consonant music of Mozart to heavy metal [24]. Birds have also been shown to prefer consonant music [56,81] but although rats can be trained to discriminate between consonance and dissonance, they do not generalize to other stimuli nor show the processing advantage that humans show to consonant versus dissonant music [82,83]. These data show that birds and primates do prefer consonant music, and it is not clear for other taxa. I suggest that consonant music be used with all music provided for enrichment.

Thus, the specific structure of the music to be used must be chosen to match the goals of those working with animals. If one is using music that is within the perceptual range of the species and music that has the specific structural features that are predicted to induce the desired behavior, then music may be used successfully [84]. In the next section, I describe research on music and behavior of animals that incorporated these points into the experimental design.

### 3.3. Effects of Species-Appropriate Music

To test these ideas, musician and composer David Teie and I have collaborated on two studies using species-appropriate music. The first study [85] tested captive cotton-top tamarins, previously shown by McDermott and Hauser [24] to prefer Mozart to heavy metal, but silence to either type of music. These monkeys are small (c.a. 500g), and in the wild live in forested habitats in Northern Colombia and use vocalizations as their main communication modality. Since the normal communication range of these animals is about three octaves higher than human speech and music and their resting heart rate is nearly three times that of an adult human, we composed the species-appropriate music with these facts in mind. We tested two examples of each of two types of music. The first set of compositions used the principles hypothesized to be involved in creating calming music—long notes, harmonic structure, lower amplitude, and slow attack speed with tempo at about the resting heart rate of tamarins. We designed the second set of compositions to be highly arousing and fear inducing with short staccato notes, dissonant or noisy features, and rapid tempos at twice the resting heart rate of tamarins. We tested two additional sets of human-based music, one pair with calming features and the other set, typical of heavy metal that would be arousing to humans. Thus, there were eight different pieces of music in total.

We tested pairs of tamarins with each of the eight musical examples, each edited to 30 s duration. Each piece was used only once with each pair in order to avoid habituation. In each session, we waited for the animals to settle after the observer entered the colony rooms and then conducted baseline observations for five minutes. Then, the observer played a 30 s. sample of music and recorded behavior for an additional five minutes. Data were analyzed by pairs since the response of one animal is not independent of its mate. We compared responses to calming versus arousing tamarin music in the five minutes after the play back, compared the five minutes baseline response to the behavior following the music, and compared responses to species-appropriate music to the similar types of human-based music.

The tamarins exhibited less motor activity and engaged in more eating and drinking behavior after the tamarin calming music, both compared with baseline behavior and compared with the arousing music. In contrast, tamarins showed increased activity, increased levels of anxiety behavior and increased huddling and grooming (social reassurance) with their mates after hearing the tamarin arousing music. In contrast, the tamarins showed few significant behavioral responses to human-based music. The only exception was some decreased locomotor behavior after being presented with human heavy metal music. This result seemed paradoxical at first, until we realized that the tempos of the human heavy metal music matched the resting heart rate of the tamarins. Thus, music that is arousing for us could actually be calming for the tamarins.

This study illustrates the two main points: the tamarins were generally uninterested and unaffected by the presentation of human-based music, showing that the musical range must match the frequency range and tempos of the species being tested. Second, the structures of music that affect different emotional responses in human listeners are also effective in nonhuman animals, if and only if, there is a perceptual match to the animal’s sensory system. Importantly, this was not a test of preference. We do not know if the animals enjoy or would avoid this music, but what we do know is that their emotional tone was affected by the music. A final observation is that most humans who have listened to this music have found it aversive. Thus, just as tamarins may find human music aversive, so humans may find tamarin music aversive.

Our second study looked at cats [86]. After the tamarin study was published, we heard from many people who use music as a putative therapy with their pets. However, as we talked with these people, we learned that each person was convinced that their pets liked the same music as they enjoyed; one person liked classical, another liked heavy metal, a third liked country music and that was what they presented to their pets. Do pets respond to human music and, if so, how? Do they find their owner’s preferred music pleasant, aversive or irrelevant?

We chose to study cats rather than dogs since the body sizes, heart rates and voices of cats are more homogeneous across breeds than dogs. The study design was similar to the previous study. Two pieces of music were composed for cats. The main theme averaged an octave higher than human music. Glides are important in natural cat vocalizations and were also incorporated in the music. The tempos of purring and suckling formed the basis of the two cat music pieces and two classical music examples were selected that were similar in structure from a human perspective. We tested cats in their own homes where we would place two speakers 1 m to the left and right of our playback computer. Each musical piece was three minutes long. We played the four selections counter-balancing the order of presentation and the side of the speaker broadcasting the music. We compared the responses of cats to the cat music compared to the human music. The cats responded with significantly shorter latency and showed significantly more interest (orienting, approaching speaker, rubbing speaker) in the cat music than in the human music. Cats that had been restless during the baseline period became calmer upon hearing the cat music. Several people have told us anecdotally in response to the paper that the cat music was especially useful with helping their shelter- and feral-adopted cats to become more relaxed and interactive with their owners. A recent study [87] compared the effects of cat music, classical music, and silence on stress-related behaviors of cats during veterinary clinic examinations and found that the cat music significantly reduced stress behavior when compared to both silence and classical music. In our study the cats were essentially indifferent to the classical music excerpts, showing neither positive nor negative reactions. Thus, there is likely no harm in playing human classical music to cats, but there is no benefit either. We do not know about the effects of other types of music.

Taken together, these studies suggest that species-appropriate music can lead to behavioral change in nonhuman animals, and that the specific features of the music can have either calming or arousing effects, so that care must be taken to assure that the music presented is structured to produce the desired results.

## 4. Discussion

Music, as we know it today, is a structurally complex system of notes, chords, tempos, themes and variations, with a complexity that some authors feel makes it similar to language [88]. However, music also evokes strong emotions (joy, sadness, anger, fear, calmness, happiness, surprise, disgust), with brain activation in areas associated with these emotions being activated by music [9,89,90]. Although the structural complexity of modern human music may have co-evolved with language, the emotional components of music may have a long evolutionary history [7]. It is likely that these emotional components have made music adaptive to humans. The induction of emotions in listeners can foster cooperation, increase social activity, create a sense of group cohesion and induce empathy. An experimental study of four-year-old children showed that children who sang music together were more likely to cooperate and help each other than children who merely recited the same words [91]. Music has also been shown to assist in healing during illness in many studies [92,93,94]. If music has this value to humans, might it not also be helpful to animals, leading to more closely integrated social groups, an ability to coordinate actions in defense? This question has led to the many studies in Table 1 evaluating music on the behavior and physiology of animals.

The vocal signals of many animals have musical components. Indeed the early studies of vocalizations before the widespread use of spectral analyses used musical notation to denote animal signals (e.g., [95]), and we call the territory defense and courtship sounds of birds “songs”. The studies of McConnell [5] and Fernald [6] on how humans use musical features to influence the behavior of working animals and nonverbal infants, respectively, provided additional impetus for the use of music with animals in managed care (zoological parks, farms, laboratories and pets). Because music written for humans is the most accessible form of music for us, it seemed quite logical to assume that human-based music would also be of benefit to animals. However, as reviewed here, it is clear that human-based music can have a variety of effects (positive, negative, and neutral) within and between species.

This variety and inconsistency of effects of music on animals can be explained in part by the use of a variety of different types of music stimuli which makes direct comparisons difficult. The mixed results may also be due to failures to realize that the vocal communication systems of animals may be fundamentally different from our own with different sensitivities to both pitch and tempo. The success of playing only the frequencies of a Mozart piece that are above 4 kHz on reducing blood pressure in hypertensive mice [48], the failure of musical manipulations that worked in chickens to have any effects in rats [57], and the ability to manipulate behavior in tamarins with music in their pitch range and tempos [85] suggest that it is important to match music to the perceptual abilities of the species.

It is equally important to understand the structural features of music that can lead to different emotional responses. Thus, it is important to articulate goals and use music that is appropriate toward achieving those goals. The music one uses to arouse an animal that appears lethargic or depressed will be very different from what one uses to calm a hyperactive animal, and, presumably, no one wants to use fear- or threat-inducing music very often. Additionally, it is important to be aware that within each musical genre there is a variety of emotions expressed musically. No one genre is uniformly calming, arousing, or threatening.

Armed with knowledge of a species’ perceptual abilities and with a clear view of the goals of playing music, one can then locate or create the music that will be best for improving the well-being of animals in human care. Those designing future research on the effects of music on animals should also pay attention to these points. Then, we will be able to say (paraphrasing William Congreve, 1670–1729) that “music hath charms to soothe (and arouse) the savage beast”.

## Figures and Tables

**Table 1:** Effects of Music and Other Sounds on Animals *.

Species	Music or Sounds	Outcome	Citation
Orangutan (Pref)	7 musical genres, silence	Silence preferred	[10]
Gorilla (Wel)	Noises, rainforest sounds	Rainforest sounds calmed infants aroused adults	[11]
Gorilla (Wel)	Classical, natural sounds, silence	No effect	[12]
Gorilla (Wel)	Classical, rock, natural sounds	Reduced stereotypy to natural sounds	[13]
Gorilla (Wel)	Classical, rock, natural sounds	No effect natural sounds, slow tempo classical, reduced anxiety	[14]
Chimpanzees (Pref)	Classical, pop rock, silence	Individual preference for pop/rock over silence	[15]
Chimpanzee (Wel)	Classical, easy listening, instrumental, vocal music	Instrumental increased affiliation, slow tempo, vocal reduced agonistic	[16]
Chimpanzee (Wel)	Unspecified music	Reduced aggression, exploration, increased social grooming, resting	[17]
Chimpanzee (Pref)	Slow tempo African, Indian, Japanese music	Apes preferred African and Indian music	[18]
Chimpanzee (Pref)	Consonant vs. dissonant	One young chimp preferred consonant music	[19]
Moloch gibbons (Pref)	Mixed classical music	No behavioral change	[20]
Lar gibbons (Pref)	Species typical songs	Increased activity, brachiation	[21]
Baboons (Phys)	Radio (Oldies)	No change in blood pressure, behavior, HR lower	[22]
Rhesus macaques Wel)	Mozart, Bach, noise, silence	Noise increased response time, not music, silence	[23]
Common marmoset (Pref)	Mozart, heavy metal, silence	Prefer Mozart to heavy metal, silence to Mozart	[24]
Cotton-top tamarin (Pref)	Mozart, heavy metal silence	Prefer Mozart to heavy metal, silence to Mozart	[24]
Elephants (Wel)	Mixed classical, silence	Classical reduced stereotypic behavior	[25]
Dogs (Wel)	Calming classical, rock, simplified classical	Classical increased sleeping, decreased vocalization, rock increased nervousness	[26]
Dogs (Wel)	Mixed classical, rock, pop, silence	Classical—more rest, decreased vocalizations	[27]
Dogs Wel, Phys)	Slow tempo classical, silence	Music increased HR variability, calmed in shelter	[28]
Dogs (Wel)	Classical music, silence	No effect on dogs but music impressed owners	[29]
Dogs (Wel)	Beethoven, pop, audiobook	Audiobook calmed shelter dogs, music did not	[30]
Lambs (Wel, Phys)	Easy music, noise, loud sounds	Noise increased weight, classical induced calm	[31]
Dairy cows (Wel)	Country music	Increased approach to milking stalls	[32]
Ponies (Wel)	Rock, classical, country, jazz	No significance, trend for country to increase eating	[33]
Piglets (Wel)	Music, pink noise, natural vocals	No effects	[34]
Piglets (Wel)	Slow tempo, fast tempo, silence	Music increased play, tail wagging	[35]
Piglets((Wel)	Slow, fast tempos, wind, strings	Fast tempos increased walking, tail wagging, slow tempos increased lying, exploration	[36]
Mice (Wel, Phys)	Perinatal Mozart, noise, silence	Mozart improved learning, increased protein levels	[37]
Rats (Wel)	Perinatal Mozart	Increased learning, frequency range not in rat range	[38]
Rats (Phys)	Ligeti versus Mozart, Different tempo, harmony	Mozart reduced HR in hypertensive rats, Ligeti increased blood pressure.	[39]
Rats (Pref, Disc)	Bach vs. Stravinsky	No preference, generalized to other similar music	[40]
Rats (Wel)	Mozart at different photoperiods	Reduced anxiety at equal photoperiod, not at others	[41]
Rats (Phys)	432 Hz tone, silence	Increased ghrelin levels, weight, dose effect	[42]
Rats (Phys)	5 types traditional Chinese music	Different neurotransmitter changes based on type of music presented	[43]
Rats (Wel)	Mozart (K448), silence	Music reduced stress and anxiety in maternal separation.	[44]
Rats (Wel, Phys)	Mozart (K488), silence	Mozart decreased fear, higher BDNF levels	[45]
Rats (Wel, Phys)	Mozart (K448), silence	Mozart enhances spatial learning high BDNF	[46]
Rats (Wel)	Mozart (K448), white noise, silence	Mozart reduced anxiety	[47]
Rats (Phys)	Mozart (K205)	Reduced BP in hypertensive rats, only frequencies over 4 kHz effective.	[48]
Hens (Wel, Phys)	Mozart vs. background noise	Music increased stress, no effect on blood measures	[49]
Hens (Pref)	Noise versus music	Noise aversive, music no effect	[50]
Chickens (Phys)	Loud noise, fast tempo raga, silence	Music increased synaptic hippocampal proteins	[51]
Chickens (Phys)	Species specific sounds, sitar music	Music increased calcium binding proteins	[52]
Chickens (Phys)	Loud noise, sitar music	Increased neuronal density, volume with music decreased density, volume with loud noise	[53]
Chickens (Phys)	Loud noise, loud music	Music enhanced auditory functions, noise did not	[54]
Chickens (Wel, Phys)	Mozart	Music reduced stress physiology, reduced growth	[55]
Chickens (Pre)	Consonant versus dissonant	Prefer consonant	[56]
Chickens (Wel, Phys)	“Happy” “sad” music	Happy music increases “happiness”, sad music increases sadness, both decrease anxiety, anger; Head flicks, feather ruffles induced by oxytocin; Increased brain norepinephrine and dopamine	[57]
Pigeons (Disc)	Bach vs. Stravinsky	Discriminated and generalized to similar composers	[58]
Java Sparrows (Disc)	Bach vs. Schoenberg	5 of 7 birds discriminated, generalized to similar	[59]
Java Sparrows (Pref)	Bach vs. Schoenberg	Two of 4 birds preferred Bach, generalized to similar composers	[60]
Carp (Phys, Wel)	Mozart, silence	Mozart increased growth rate, decreased stress	[61]
Carp (Phys)	Mozart (K529), Romanza, silence	Both music pieces increased growth, Mozart less	[62]
Seabream (Phys)	Mozart (K 529), silence	Mozart increased growth rate, energy utilization	[63]
Goldfish (Phys)	Lute violin music	No effects on growth or weight	[64]
Goldfish (Disc, Pref)	Bach vs. Stravinsky	Discriminate between but prefer neither	[65]
Turbot (Phys)	Music at different tempos	Growth improved with slow, impaired with fast	[66]

* Study outcome goals: (Pref = preference; Disc = discrimination; Wel = welfare; Phys = physiology).

**Table 2:** How Music Conveys Emotions.

Variable	Calming	Arousing	Fear	Threat
Tempo	Slow	Fast	Fast	Moderate
Pitch	Descending	Ascending	High	Low
Rate	Legato	Staccato	Staccato	Staccato
Amplitude	Soft	Loud	Loud	Moderate
Harmony	Consonant	Consonant	Dissonant	Dissonant
Attack Speed	Slow	Fast	Slow	Fast

Adapted from [80].

## Data Availability

Data is contained within the article.
